# Tinnitus Prevalence, Associated Characteristics, and Treatment Patterns among Adults in Saudi Arabia

**DOI:** 10.3390/audiolres14050064

**Published:** 2024-09-01

**Authors:** Ahmad A. Alanazi

**Affiliations:** 1 Department of Audiology and Speech Pathology, College of Applied Medical Sciences, King Saud bin Abdulaziz University for Health Sciences, Riyadh 11481, Saudi Arabia; alanaziahm@ksau-hs.edu.sa; 2 King Abdullah International Medical Research Center, Riyadh 11481, Saudi Arabia; 3 Audiology Clinic, ENT Division, Department of Surgery, King Abdulaziz Medical City, Ministry of National Guard Health Affairs, Riyadh 11426, Saudi Arabia

**Keywords:** awareness, etiology, hearing loss, management, quality of life, Saudi Arabia, severity, tinnitus

## Abstract

Tinnitus affects millions of people around the world and causes significant negative impacts on their quality of life (QoL). Tinnitus is rarely examined in Saudi Arabia. This study aimed to estimate the prevalence of tinnitus among adults, explore their experience with tinnitus, investigate the impact of tinnitus on their QoL, and discover their tinnitus management methods. A descriptive cross-sectional study design was performed utilizing a non-probability purposive sampling technique and a face-to-face in-person administered questionnaire. Descriptive statistics and a chi-square test were used to assess the data and find any correlation between the variables. Out of 4860 adults, 320 (males: *n* = 172; females: *n* = 148; age range = 18–90 years) had tinnitus, mainly described as a daily, gradual, continuous, whistling, and ringing tinnitus in both ears. Tinnitus prevalence was estimated at 6.54% with a slight predominance in males (6.9%) compared with females (6.2%). Most of the participants were unaware of the cause of their tinnitus. The modal value of the severity of tinnitus signals was severe for both genders. The modal value of the impact of tinnitus on the QoL was moderate for males and severe for females. Sleep, social activities, quiet settings, and concentration were largely affected by tinnitus. Significant associations (*p* < 0.05) between the impact of tinnitus on the QoL and risk factors, such as gender, age, hearing loss, and hyperacusis were determined. Also, the impact of tinnitus on the QoL was significantly associated (*p* < 0.05) with the duration of complaints and the severity of tinnitus signals. Approximately, 61% of the participants did not use any tinnitus treatment, while the remaining participants usually used hearing aids, medications, and counseling to manage their tinnitus. By increasing awareness, establishing standard practice, developing guidelines for managing tinnitus, expanding access to suitable interventions, and carrying out additional research, adults living with tinnitus in Saudi Arabia will have better support and, ultimately, an enhancement of their overall well-being.

## 1. Introduction

Tinnitus is the sensation of sounds without the presence of an external sound source [1]. It is often described as a ringing, buzzing, whistling, hissing, or pulsatile sound in the ears and/or head and can be continuous or intermittent [2,3]. The cause of tinnitus may be idiopathic or indirectly connected with risk factors, such as gender, age, hearing loss, exposure to loud noises, certain diseases and medications, and head trauma [4,5]. Therefore, tinnitus is not considered a disorder, but a symptom of a variety of health conditions [6]. It becomes a disorder when associated with suffering, such as autonomic arousal, emotional distress, cognitive dysfunction, and insomnia [7,8,9]. Therefore, the quality of life (QoL), the extent to which a person may enjoy life while maintaining their well-being, is significantly lowered by tinnitus [10]. Tinnitus is classified as a chronic condition if its symptoms last for longer than six months [11]. The existence of tinnitus, however, does not always indicate that there is suffering involved [12].

Tinnitus is frequently measured by subjective rather than objective methods, such as the tinnitus handicap inventory (THI) and the tinnitus functional index (TFI) [13,14]. To improve diagnosis, objective measures of tinnitus are urgently required [15]. Although there are various approaches to managing tinnitus, such as hearing aids, tinnitus retraining therapy (TRT), and cognitive behavioral therapy (CBT), a permanent cure for tinnitus has not yet been discovered due to the wide range of associated etiologies and pathogenic mechanisms of tinnitus [16]. The current treatment strategies aim to control the underlying causes and symptoms and suppress the perception of tinnitus [16].

The number of tinnitus sufferers worldwide is large, with different estimated prevalences. The prevalence of tinnitus among adults ranged from 4.1% to 37.2% [17]. However, tinnitus is rarely examined in Saudi Arabia, and its prevalence is unknown [18]. Only limited studies investigated tinnitus and its impact on the QoL of adults in Saudi Arabia [18,19]. For example, Alsanosi explored the impact of tinnitus on the QoL among 100 tinnitus patients aged 34–60 years. Tinnitus was found to be more common among men than women, and tinnitus effects were significantly higher in patients with associated hearing loss compared to those with no hearing loss [18]. Musleh et al. also examined the impact of tinnitus on the QoL among 163 adults aged 18–65 years. Their study revealed significant correlations between sociodemographic variables (e.g., smoking habits) and the impacts of tinnitus on the QoL and emotional well-being [19]. The current study aimed to address the gap in the literature by estimating the prevalence of tinnitus among adults in Saudi Arabia, exploring their experience with tinnitus, examining how tinnitus affected their QoL, and identifying what management strategies were used.

## 2. Materials and Methods

This cross-sectional descriptive study was approved by the King Abdullah International Medical Research Center Institutional Review Board under protocol #NRC23R/639/09. The procedures adhered to the ethical guidelines of the Declaration of Helsinki. Written informed consent was obtained from all the participants in this study.

### 2.1. Design of Questionnaire

A questionnaire was prepared to elicit information on tinnitus among adults in Saudi Arabia based on modified questions from previous research because of the lack of standardized similar questions [20,21]. Most questionnaires are commonly prepared in the English language and must be translated and validated in other languages [22]. The questionnaire was initially prepared by the author in the English language to capture the following four sections: demographics and associated factors, experience of tinnitus, impact of tinnitus, and management of tinnitus. Two clinical audiologists with several years of experience validated the questions. The necessary corrections suggested by the experienced audiologists were incorporated during the finalization of the survey questions. The original English questionnaire was translated into Arabic by two independent professionals who are bilingual in both Arabic and English. Subsequently, both experts recognized and addressed any deficiency in the translational ideas. Then, the Arabic version was translated back into English.

### 2.2. The Pilot Questionnaire

Hard copies of the pilot questionnaire were randomly given to twelve participants, who were required to read and fill out an informed consent form before completing the questionnaire. The participants were questioned regarding the clarity of each question to make sure there was no misinterpretation. All participants in this part reported that the questionnaire used simple and unambiguous language. Additionally, the reliability of the questionnaires was validated using Cronbach’s alpha. A score of 0.81 was obtained (α > 0.70 is the accepted cut-off point for high internal consistency). The sample size of the main study did not include those twelve participants.

### 2.3. The Main Questionnaire

The final version of the questionnaire consisted of 21 questions divided into four sections (Appendix A). Out of twenty-one questions, (a) six questions were related to the demographics of participants and associated factors, including gender, age, nationality, hearing status, hearing aid/s usage, and sound tolerance, (b) eight questions were related to their experience with tinnitus, including cause of tinnitus, period of tinnitus complaints, onset of tinnitus, duration of tinnitus, perception time of tinnitus, type of tinnitus, location of tinnitus, and severity of tinnitus signals, (c) four questions focused on the impact of tinnitus, including the effect of tinnitus on their QoL, fear of tinnitus getting worse, feeling depressed or anxious because of tinnitus, and types of activities affected by tinnitus, and (d) three questions were about the management of tinnitus, including what type of management was used, their satisfaction with it, and whether further information about tinnitus was needed. An ordinal scale of 1–10, with 10 representing very severe, was used to help the participants rank the severity of tinnitus signals and the impact of tinnitus on their QoL. The ordinal scale was applied for the following questions: what is the severity of your tinnitus signals? and what is the impact of tinnitus on your QoL?. Their responses were divided into three groups: mild tinnitus signals/mild impact (1–4), moderate tinnitus signals/moderate impact (5–7), and severe tinnitus signals/severe impact (8–10).

### 2.4. Participants

Adult patients (*n* = 4860) who visited the audiology clinic at King Abdulaziz Medical City in Riyadh, Saudi Arabia, from November 2023 to June 2024 were the target population. Most of them visited the clinic for the first time and were mostly referred from the primary healthcare centers due to decreased hearing sensitivity, vertigo, dizziness, and/or tinnitus with other audiological concerns, such as increased sensitivity to sounds, otalgia, and aural fullness. Other patients visited the clinic for an annual hearing assessment, hearing aid fitting, or hearing aid follow-up. All patients who had tinnitus and agreed to consent were included.

### 2.5. Data Collection and Analysis

A non-probability purposive sampling technique was used. All included participants, who visited the clinic for the first time or the annual hearing assessment, underwent case history taking, otoscopy examination, tympanometry, pure-tone audiometry, and speech audiometry as per the clinic protocol. Acoustic reflexes and otoacoustic emissions were performed based on the case and the results of other assessments. The questionnaire was administered before the participants were counseled about tinnitus to avoid any bias that could affect their responses to the questions (e.g., do you know the cause of your tinnitus?). The participants were verbally asked, and their responses were recorded on printed hard copies of the questionnaire and attached to the signed consent form for each participant. All the responses to the questionnaire were converted into a numerical form using Microsoft Excel. Statistical Package of Social Science (SPSS v.26) software was used to analyze the collected data using descriptive and inferential statistics. Descriptive statistics were used to present socio-demographic data for categorical variables, which included frequency and percentages. The chi-square test was used to find the association between the impact of tinnitus on the QoL, gender, age, duration of complaints, severity of tinnitus signals, hearing loss, and hyperacusis. A *p*-value less than 0.05 was considered statistically significant.

## 3. Results

### 3.1. Prevalence of Tinnitus and Associated Factors

Data were collected from 320 tinnitus participants (males: *n* = 172; females: *n* = 148) with ages ranging from 18 to 90 years (Table 1). Nearly 70.6% of the participants were aged between 51 and 80 years. Most of the participants were Saudis, comprising 98.8% of the total. The prevalence of tinnitus was estimated at 6.58% (95% CI, 5.9–7.3). The prevalences of tinnitus among male and female participants were 6.9% (95% CI, 5.9–8.0) and 6.2% (95% CI, 5.2–7.2), respectively. Of the total participants, 223 (69.7%) were diagnosed with hearing loss, which was mainly sensorineural hearing loss (82.1%). Only 61 (19.1%) participants used hearing aids. When the participants were asked about their loudness perception in response to auditory stimuli of normal volume, 171 (53.4%) stated normal sound tolerance (Table 1).

### 3.2. Experience of Tinnitus

Table 2 summarizes the experience of tinnitus among the participants. Of the total (*n* = 320), 205 (64%) were not aware of the cause of their tinnitus. The most common reported causes of tinnitus by the remaining participants were noise exposure (43.1%) and ear-related problems (35.4%), including hearing loss, ear infections, and tympanic membrane perforations. The participants complained of tinnitus for different periods, of which more than ten years (27.8%) and one to two years (23.8%) were mostly stated. The results revealed that 169 (52.8%) of the participants stated a gradual onset of tinnitus, while 151 (47.2%) reported a sudden onset. The majority of participants stated a daily, continuous tinnitus. Whistling (43.4%), ringing (39.7%), and whooshing (27.2%) were the common types of tinnitus described by the participants, respectively. Approximately half of the participants experienced tinnitus in both ears, followed by left ear (23.1%), right ear (18.8%), and head (14.4%).

**Figure 1:**
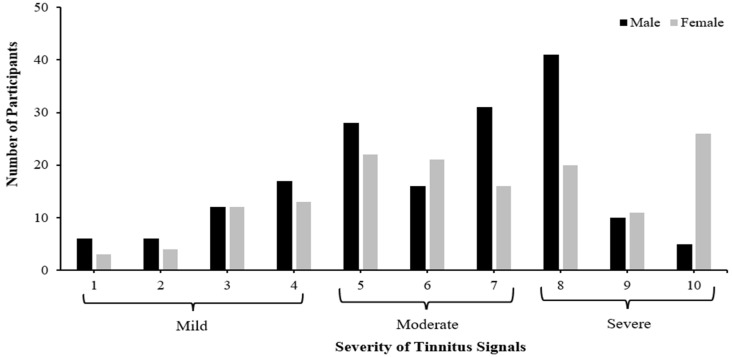
The number of participants (*n* = 320; males = 172; females = 148) and the severity of their tinnitus signals.

Figure 1 displays the severity of tinnitus signals for both male and female participants. Most of the male participants reported their tinnitus as moderate (34.3%) and severe (33.1%), respectively. The modal average of the severity of their tinnitus signals was eight (severe). Similarly, most of the female participants reported their tinnitus as moderate (50.7%) and severe (38.5%), respectively. The modal average of the severity of their tinnitus signals was ten (severe).

### 3.3. Impact of Tinnitus

Of the total participants, 198 (61.9%) reported being afraid that their tinnitus may get worse. Tinnitus caused depression or anxiety for 139 (43.4%) participants. Most participants (86.3%) reported that tinnitus negatively affected their daily activities. Sleep (56.6%), social activities (46,3%), quiet settings (44.4%), concentration (41.3%), work (12.2%), and sports (6.9%) were mostly affected by tinnitus, respectively.

**Figure 2:**
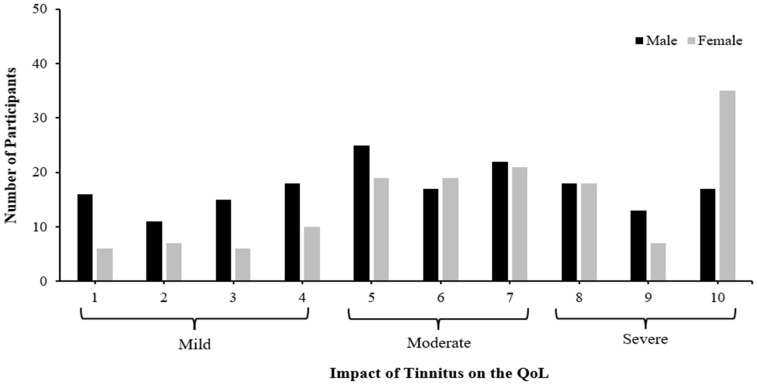
The number of participants (*n* = 320; males = 172; females = 148) and the impact of tinnitus on their quality of life (QoL).

Figure 2 depicts the impact of tinnitus on the QoL for both male and female participants. Most of the male participants reported that their tinnitus had mild (37.2%) and moderate (34.9%) impacts on their QoL, respectively. The modal average of the impact of tinnitus on their QoL was five (moderate). On the other hand, most of the female participants reported that their tinnitus had severe (40.5%) and mild (39.9%) impacts on their QoL, respectively. The modal average of the impact of tinnitus on their QoL was ten (severe).

### 3.4. Management of Tinnitus

**Figure 3:**
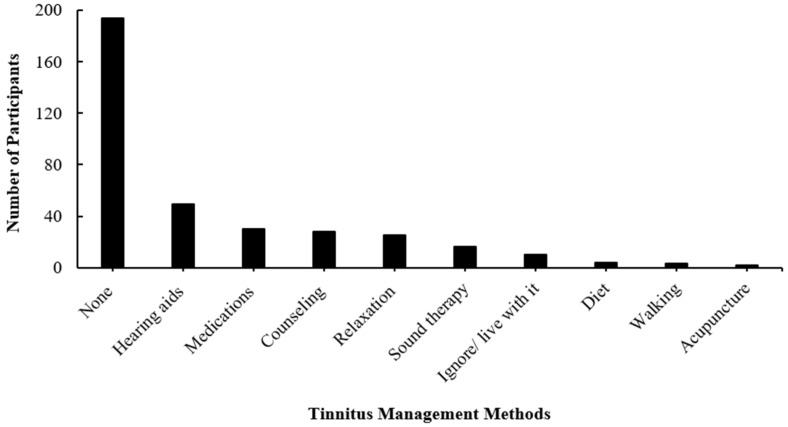
The number of participants (*n* = 320) and their tinnitus management methods. Note, more than one option was allowed for selection.

Figure 3 shows the type of tinnitus management used by the participants. Unsurprisingly, most of the participants (60.6%) did not use any management for their tinnitus. The remaining participants used hearing aids (15.3%), medications (9.3%), consultation (8.7%), relaxation (7.8%), and sound therapy (5%) as tinnitus management methods, respectively. Learning to ignore tinnitus was also used as a tinnitus management technique by nearly 3% of the participants. ‘Not applicable’ was mostly chosen by the participants to respond to the question about their satisfaction with the management method they used for tinnitus because most of them did not use any. Two hundred and ninety-six participants (92.5%) requested more information about tinnitus.

### 3.5. Association between the Impact of Tinnitus and Other Variables

Table 3 summarizes the association between the impact of tinnitus on the QoL, gender, age, duration of complaints, severity of tinnitus signals, hearing loss, and hyperacusis. A significant association (*p* = 0.005) was found between the impact of tinnitus on the QoL and gender. Male participants tended to have a moderate impact, while female participants tended to have a severe impact. The results showed that the impact of tinnitus on the QoL increased with age. Tinnitus severely impacted the QoL of participants who were older than 70 years. The impact of tinnitus on the QoL was significantly associated with age (*p* = 0.001). The duration of complaints varied among all participants and was distributed across all the categories of the impact of tinnitus. The impact of tinnitus on the QoL was significantly associated with the duration of complaints (*p* = 0.001). The association between the impact of tinnitus on the QoL and the severity of tinnitus signals (*p* = 0.001) was significant. When the severity of tinnitus signals increased, the impact of tinnitus on the QoL increased accordingly. Interestingly, more than half of the participants with hearing loss had a mild impact of tinnitus on their QoL, and the QoL of the participants who had normal hearing was either moderately or severely affected. The results revealed a significant association between the impact of tinnitus on the QoL and hearing loss (*p* = 0.001). The results showed that the QoL was severely affected among 71.6% of the participants who had hyperacusis, while a mild impact on the QoL was found among 65% of those who did not have hyperacusis. A significant association (*p* = 0.001) between the impact of tinnitus on the QoL and hyperacusis was determined.

## 4. Discussion

Tinnitus is a common, debilitating health condition that remains a mystery because of various etiologies, complicated pathogenic mechanisms, and the lack of a definitive cure. The present study explored the prevalence, experience, impact, and management of tinnitus among adults in Saudi Arabia.

### 4.1. Prevalence of Tinnitus and Associated Factors

The prevalence of tinnitus among adults in Saudi Arabia was found to be 6.58%. This prevalence falls within the range of worldwide prevalence of tinnitus [17]. It is higher than tinnitus prevalences in neighboring countries, such as Iran (4.6%) [23] and Egypt (5.17%) [24], and lower than tinnitus prevalences in other neighboring countries, such as the United Arab Emirates (19.3%) [25] and Jordan (28.8%) [26]. This variation in prevalence may be attributed to differences in the survey questions, age of participants, and statistical methods used in these studies. The prevalence of tinnitus among males in our study was 6.9%, while it was 6.2% among female participants. Contrasting evidence to support a gender difference in tinnitus prevalence exists [27]. Several studies found higher prevalence in males [18,28], in females [29,30], and a similar prevalence among both genders [17,31]. 

Interestingly, nearly two-thirds of the participants were unaware of the cause of their tinnitus. Noise exposure and ear-related problems were the main reported possible causes of tinnitus by the remaining participants. Tinnitus is frequently associated with two prevalent types of hearing loss: noise-induced hearing loss (NIHL) and age-related hearing loss (i.e., presbycusis) [32,33]. However, it is possible to have tinnitus without any identifiable hearing loss or hearing loss without any tinnitus [34]. Exposure to loud noises among males, rather than females, was assumed to be the reason for the gender difference in the prevalence of tinnitus [35]. Our study included active and retired military personnel who were regularly exposed to hazardous levels of noise in their work. Only 50 participants reported noise exposure as the probable cause of their tinnitus. The prevalence of NIHL among Saudi military personnel (71.6%) is high due to the lack of awareness of NIHL and improper use of hearing protection [36]. Hearing protection programs are advised to be established to raise awareness, protect hearing, and eliminate NIHL consequences, including tinnitus [36]. Presbycusis is common in Saudi Arabia, and accordingly, tinnitus may be more prevalent in people who have hearing impairments [37]. Our study showed that about 73% of the participants were older than 50 years, and 70% of them had hearing loss. Sensorineural hearing loss was the predominant type of hearing loss. Our finding that tinnitus is common among the elderly has been consistently reported in the literature [30,38].

Nearly half of the participants in our study reported hyperacusis. Like hearing loss, tinnitus is highly associated with hyperacusis [34]. About 90% of people with hyperacusis report coexisting tinnitus [39]. The relationship between tinnitus and hyperacusis increases with severity reaching 80% [40]. Only four participants reported that medications might be the potential cause of their tinnitus. Tinnitus is a known side effect of many drugs, such as non-steroidal anti-inflammatory drugs (NSAIDs), aminoglycosides antibiotics, and loop diuretics [41,42]. For example, NSAIDs are usually used as a medication for treating patients with osteoarthritis [42], which is a common health condition that develops with increasing age in Saudi Arabia [43]. Stress was also reported as a cause of tinnitus by a few participants in our study. Tinnitus is frequently linked to stress, and patients frequently claim that stressful events exacerbate their tinnitus [44]. These reported causes of tinnitus are modifiable and can be controlled to decrease the prevalence of tinnitus in Saudi Arabia.

Based on the identified lack of awareness about tinnitus and its causes among the participants, awareness campaigns are considered critical in tinnitus awareness, hearing loss prevention, and health promotion generally. Unfortunately, there have been only a few awareness campaigns regarding the value of hearing screening and the negative effects of hearing loss in Saudi Arabia [45]. Although awareness about tinnitus can come from Internet sources and may be more beneficial [46,47], elderly people may be unable to use technology and access web-based health information sources because of several factors, such as a lack of digital literacy and high costs [48].

### 4.2. Experience of Tinnitus

Different aspects of tinnitus were reported by the participants. Tinnitus was continuously perceived daily for a few years with a slight predominance of gradual onset compared with sudden onset of tinnitus. Gradual tinnitus can be a result of presbycusis and noise exposure for long durations [32,37], while sudden tinnitus can occur because of acoustic trauma [49], sudden sensorineural hearing loss [50], and ototoxicity [51]. Presbycusis and NIHL were commonly reported by the participants in our study. These factors, in addition to chronic diseases and the use of medications, usually affect both ears and cause tinnitus [26]. This may also explain the higher percentage of bilateral tinnitus reported by the participants. Approximately 30% of the participants had intermittent tinnitus, which may be related to excessive noise exposure [52].

It is important to identify if tinnitus is pulsatile or non-pulsatile because of different etiologies and management methods [53]. Tinnitus is always described as ‘ringing in the ear’ regardless of the sound of tinnitus [54]. Sounds of tinnitus, such as whistling, ringing, and whooshing were stated by the participants in our study. Ringing tinnitus is related to hearing loss caused by loud exposure [54], while whooshing tinnitus can be caused by turbulent blood flow in the vessels close to the ear [55]. Pulsatile tinnitus was reported by nearly 8% of the participants. This type of tinnitus is an indication of probable vascular pathologies [56].

Our findings showed that ‘severe’ was the modal average of the severity of tinnitus signals for both male and female participants. However, tinnitus intensity may not always accurately reflect how the condition affects a person’s daily life [57]. Even someone with a mild severity of tinnitus signals may occasionally feel as though it significantly affects their day-to-day activities, while a person with particularly severe tinnitus signals might believe that it has little to no effect on day-to-day activities [58]. Therefore, it is important to distinguish between the severity of tinnitus signals and the severity of tinnitus (i.e., the impact of tinnitus) on life, which is more important [59].

### 4.3. Impact of Tinnitus

Living with tinnitus can have a serious impact on an individual’s QoL. The constant presence of tinnitus in the ears can significantly affect a person’s physical health, mental well-being, and social interactions. Recently, “tinnitus disorder” was recommended to describe the auditory component and the associated suffering, while “tinnitus” was limited to defining the auditory component [7]. Therefore, the QoL is predicted by tinnitus-associated suffering rather than the psychophysical measures of tinnitus [59]. Nearly 62% of the participants in our study were afraid of deteriorating tinnitus. A lower QoL is related to a higher level of fear of tinnitus [59]. Less than half of the participants were depressed or anxious about their tinnitus. The perception of tinnitus does not always cause distress or anxiety, but it can have profound effects on the QoL [34]. Generally, tinnitus patients are more likely to have depression [60]. It needs to be taken into consideration that many tinnitus patients experience depression and anxiety before tinnitus, which is not always the starting point of tinnitus-related problems [61]. The participants reported that sleep, social activities, quiet settings, and concentration were mostly affected by tinnitus. Our results are in accordance with previous findings in studies that showed many of the tinnitus effects are psychological rather than physical [54,62,63]. 

Higher levels of the impact of tinnitus on the QoL were found for females and older participants, with long periods of complaints and severe tinnitus signals in the presence of hearing loss and hyperacusis. The modal average of the impact of tinnitus on the QoL was moderate in males and severe in females. Females are more burdened and affected by tinnitus than males [23]. More than half of the participants experienced daily, constant tinnitus in both ears, so they were at a higher risk of a poorer QoL. Patients with binaural tinnitus have more significant sleep disturbances than those with tinnitus in only one ear [64]. This also explains why the severity of tinnitus signals and the impact of tinnitus on the QoL scores were high among the participants. Our study found a significant association between age and the impact of tinnitus on the QoL that was similar to other studies [65,66]. In contrast, other studies found no association between age and the effects of tinnitus [19,67]. 

The current study showed that a tinnitus duration of one to two years was associated with moderate to severe impacts on the QoL. This indicates that the effect of tinnitus on the QoL is vital during the first years of tinnitus. Although habituation and acceptance of tinnitus tend to increase over time and decrease tinnitus annoyance [68], we found the impact of tinnitus on the QoL was high after a few years since the beginning of tinnitus. This might show evidence that habituation has not occurred. Hearing loss and hyperacusis were common among the participants. Both hearing loss and hyperacusis, whenever present with tinnitus, are associated with greater impacts on the QoL. Tinnitus, hearing loss, and hyperacusis are seen as a triad because of the pathophysiologic mechanisms [69]. Hearing loss and hyperacusis affect how tinnitus is perceived [70]. A higher loss of the QoL and distress were seen in patients with tinnitus and hyperacusis [71,72,73].

### 4.4. Management of Tinnitus

Tinnitus management is usually designed to address the specific needs of each patient because each patient has a unique medical, social, and/or psychological history. No medication has been approved as a treatment for tinnitus, but a variety of methods are currently used to control rather than completely cure tinnitus and improve the QoL [16,74]. Hearing aids, sound therapy, relaxation therapy, CBT, and TRT are some of these management methods [75]. The present study showed that 60% of the participants did not use any method to manage tinnitus. The lack of knowledge about tinnitus, its causes, and management options could be the reason [76]. A limited number of management methods were used by the remaining participants. Hearing aids were commonly used among the participants, though only 22% of the participants with hearing loss used hearing aids. This might be because many of them visited the clinic for the first time and were not seen in other audiology clinics. The reduction in hearing disability through hearing aid fitting has a positive effect on tinnitus [77]. Hearing aids can improve tinnitus-related distress and speech comprehension, but audiological and psychological variables may influence the use time [78]. Medications were used for managing tinnitus by some of the participants. Although there is no single approved drug for treating tinnitus in the market, medications prescribed for treating depression, anxiety, and insomnia have been used to treat tinnitus [41,79]. Other medications (e.g., antibiotics and hypertensives) may alleviate tinnitus by treating associated conditions, such as ear infections or hypertension [16].

Counseling was also selected as a method for managing tinnitus by only 28 participants. Counseling provides positive information to remove any fears or anxieties that are associated with tinnitus [80]. Negative information frequently provided to tinnitus sufferers can worsen the condition [81]. Therefore, healthcare professionals (e.g., audiologists and otorhinolaryngologists) who deal with tinnitus patients should avoid the use of negative consultation, such as tinnitus is untreatable and little is known about it. Most tinnitus patients, when they know there is no cure for tinnitus, would struggle to cope [82]. Some of the participants in the current study were using a variety of coping techniques to manage their tinnitus and compensate for the lack of cure, such as diet, acupuncture, relaxation, and walking. CBT is seen as the most effective treatment for tinnitus [34]. However, neither CBT nor TRT were stated for managing tinnitus by the participants. Tinnitus services are limited in Saudi Arabia [83].

Most participants (92.5%) wanted additional information about tinnitus. All patients have the consistent desire to access reliable information regarding their health condition, but patients at early stages of their health condition onset have a greater need for information [84]. This information can be provided through tinnitus information sessions, which play an important role in educating patients about tinnitus and empower them to take steps to habituate to their tinnitus [82]. During these sessions, tinnitus patients are provided with comprehensive information about tinnitus and management methods to reduce their fear of illness. Information sessions are beneficial for patients with chronic conditions, such as diabetes and multiple sclerosis [84,85].

## 5. Study Limitations and Future Research

Efforts were made to minimize study limitations, though some were unavoidable. This study relied on data that were highly representative of the target population; however, it included adults who visited one audiology clinic. Therefore, the interpretation and overgeneralization of the results should be taken with caution. Information recall bias might affect the participants’ responses to some questions. For example, the question ‘How long have you had tinnitus?’ determines the length of time that the attendee has been consciously aware of the presence of tinnitus. The answers to this question may not be 100% accurate because tinnitus could be present for a long period, but the reactions to it may have started recently. Further research is needed to better understand specific demographics and the associated risk factors contributing to tinnitus in Saudi Arabia. Longitudinal studies can provide insights into the progression of tinnitus and its impact on different aspects of individuals’ lives. Further research can also investigate various age groups and prevalence across these groups in different regions in Saudi Arabia. Additionally, examining the effectiveness of various management and treatment options will help develop tailored interventions that address the unique needs of adults with tinnitus in Saudi Arabia.

## 6. Conclusions

Tinnitus is a prevalent health condition that affects a significant number of adults in Saudi Arabia, and it deserves attention. This study showed the negative impact of tinnitus on the QoL of adults and the limited use of tinnitus management methods in Saudi Arabia. Tinnitus patients should have available accurate, up-to-date, and comprehensive information about tinnitus and the existing methods that could assist with managing their tinnitus. Public health campaigns can promote preventive measures, such as hearing protection and noise reduction, to minimize the risk of developing tinnitus. There is an urgent need to establish standard practice and prepare guidelines for managing tinnitus in all audiology clinics in Saudi Arabia. Professional organizations, tinnitus researchers, and clinicians should work as a team to establish these standards and guidelines.

## Figures and Tables

**Table 1:** Demographics of the participants and their associated factors.

Variable	Total (*n* = 320)		
	*n* (%)		
Gender			
Male	172 (53.8)		
Female	148 (46.2)		
Age (years)	Male	Female	Total
18–30	13 (7.5)	14 (9.5)	27 (8.4)
31–40	12 (7.0)	11 (7.4)	23 (7.2)
41–50	23 (13.4)	14 (9.5)	37 (11.6)
51–60	39 (22.7)	34 (23.0)	73 (22.8)
61–70	50 (29.1)	43 (29.0)	93 (29.1)
71–80	31 (18.0)	29 (19.6)	60 (18.7)
81–90	4 (2.3)	3 (2.0)	7 (2.2)
>90	0 (0.0)	0 (0.0)	0 (0.0)
Nationality			
Saudi	316 (98.8)		
Non-Saudi	4 (1.2)		
Hearing loss			
Yes	223 (69.7)		
No	97 (30.3)		
Hearing aid/s usage			
Yes	61 (19.1)		
No	259 (80.9)		
Sound tolerance			
Normal	171 (53.4)		
Abnormal (Hyperacusis)	149 (46.6)		

**Table 2:** Experience of tinnitus among the participants.

Experience of Tinnitus	Total (*n* = 320)
	*n* (%)
Cause	
Unaware	205 (64)
Aware	116 (36)
	Probable causes
	Noise exposure: 50 (43.1) Ear-related problems: 41 (35.4) Head and/or neck trauma: 13 (11.2) Stress and anxiety: 5 (4.3) Medications: 4 (3.4) High blood pressure: 3 (2.6)
Period of complaints (years)	
<1	42 (13.1)
1–2	76 (23.8)
3–5	58 (18.1)
6–8	22 (6.9)
9–10	33 (10.3)
>10	89 (27.8)
Onset	
Gradual	169 (52.8)
Sudden	151 (47.2)
Duration	
Continuous	217 (67.8)
Intermittent	103 (32.2)
Perception time	
Daily	227 (70.9)
Weekly	37 (11.6)
Monthly	6 (1.9)
Sometimes	50 (15.6)
Type *	
Whistling	139 (43.4)
Ringing	127 (39.7)
Whooshing	87 (27.2)
Pulsing	26 (8.1)
Buzzing	9 (2.8)
Clicking	3 (0.9)
Location *	
Both ears	166 (51.9)
Left ear	74 (23.1)
Right ear	60 (18.8)
Head	46 (14.4)

* More than one option was allowed for selection.

**Table 3:** The association between the impact of tinnitus on the quality of life and gender, age, duration of complaints, severity of tinnitus, and hearing loss.

Variables	Impact of Tinnitus on the QoL (*n* = 320)			
	Mild Impact * n (%)	Moderate Impact * n (%)	Severe Impact * n (%)	*p*-Value ^+^ (Chi-Square Test)
Gender				
Male	60 (67.4)	64 (52)	48 (44.4)	0.005
Female	29 (32.6)	59 (48)	60 (55.6)	
Age (years)				
18–30	27 (22.0)	0	0	0.001
31–40	23 (18.7)	0	0	
41–50	36 (29.3)	0	0	
51–60	37 (30.0)	36 (40.4)	0	
61–70	0	53 (59.6)	41 (38.0)	
>70	0	0	67 (62.0)	
Period of complaints (years)				
<1	42 (34.2)	0	0	0.001
1–2	0	28 (31.5)	48 (44.4)	
3–5	41 (33.3)	0	17 (15.8)	
6–8	22 (17.9)	0	0	
9–10	1 (0.8)	32 (35.9)	0	
>10	17 (13.8)	29 (32.6)	43 (39.8)	
Severity of tinnitus signals				
Mild	73 (59.3)	0	0	0.001
Moderate	50 (40.7)	81 (91.0)	3 (2.8)	
Severe	0	8 (9.0)	105 (97.2)	
Hearing loss				
Yes	123 (100)	40 (44.9)	60 (55.6)	0.001
No	0	49 (55.1)	48 (44.4)	
Hyperacusis				
Yes	57 (35)	29 (42)	63 (71.6)	0.001
No	106 (65)	40 (58)	25 (28.4)	

* Based on the ordinal scale (1–10): mild impact (1–4), moderate impact (5–7), and severe impact (8–10). ^+^ Statistically significant at a less than 5% level of significance. QoL: Quality of life.

## Data Availability

The data presented in this article are available from the corresponding author upon reasonable request.

## Appendix A. Questionnaire

A. **Demographics and associated factors** 
  1. What is your gender?
    ○ Male
    ○ Female
  2. What is your age (years)?
    ○ 18–30
    ○ 31–40
    ○ 41–50
    ○ 51–60
    ○ 61–70
    ○ 71–80
    ○ 81–90
    ○ >90
  3. What is your nationality?
    ○ Saudi
    ○ Non-Saudi
  4. Do you have hearing loss based on a hearing test conducted by an audiologist?
    ○ Yes
    ○ No
  5. Do you wear a hearing aid/s?
    ○ Yes
    ○ No
  6. Does your reaction to any sound (sound tolerance) similar to people around you?
    ○ Yes
    ○ No (please specify)
B. **Experience of Tinnitus** 
  7. Do you know the cause of your tinnitus?
    ○ Yes (please specify)
    ○ No
  8. How long have you had tinnitus (years)?
    ○ <1
    ○ 1–2
    ○ 3–5
    ○ 6–8
    ○ 9–10
    ○ >10
  9. What is the first onset of your tinnitus?
    ○ Sudden
    ○ Gradual
  10. How do you describe the duration of your tinnitus?
    ○ Constant
    ○ Intermittent
  11. What is the number of times you perceive tinnitus?
    ○ Daily
    ○ Weekly
    ○ Monthly
    ○ Sometimes
    ○ Other (please specify)
  12. What is the sound of your tinnitus? (Choose all applicable)
    ○ Pulsing
    ○ Ringing
    ○ Whooshing
    ○ Whistling
    ○ Buzzing
    ○ Clicking
    ○ Other (please specify)
  13. Where do perceive your tinnitus? (Choose all applicable)
    ○ Right Ear
    ○ Left Ear
    ○ Both Ears
    ○ Head
  14. What is the severity of your tinnitus? (Choose from 1 to 10, [10 is very severe])
    ○ 1
    ○ 2
    ○ 3
    ○ 4
    ○ 5
    ○ 6
    ○ 7
    ○ 8
    ○ 9
    ○ 10
C. **Impact of Tinnitus** 
  15. What is the impact of tinnitus on your quality of life? (Choose from 1 to 10, [10 is very severe])
    ○ 1
    ○ 2
    ○ 3
    ○ 4
    ○ 5
    ○ 6
    ○ 7
    ○ 8
    ○ 9
    ○ 10
  16. Are you afraid that your tinnitus gets worse?
    ○ Yes
    ○ No
  17. Does your tinnitus make you feel depressed or anxious?
    ○ Yes
    ○ No
  18. What activities are affected by your tinnitus? (Choose all applicable)
    ○ Concentration
    ○ Work
    ○ Sleep
    ○ Social
    ○ Sports
    ○ Quiet settings
    ○ Other (please specify)
D. **Management of Tinnitus** 
  19. What management/treatment services and/or devices have you tried to manage your tinnitus? (Choose all applicable)
    ○ Hearing aids
    ○ Sound therapy
    ○ Medical Counseling
    ○ Medications
    ○ Relaxation
    ○ Cognitive Behavioral Therapy
    ○ Tinnitus Retraining Therapy
    ○ Other (please specify)
    ○ Nothing
  20. Are you satisfied with the method you have been using to relieve your tinnitus?
    ○ Yes
    ○ No
    ○ Not applicable
  21. Would you like to receive more information about tinnitus?
    ○ Yes
    ○ No
